# Effect of low-level laser physiotherapy on left ventricular function among patients with chronic systolic heart failure

**DOI:** 10.1186/s43044-023-00337-6

**Published:** 2023-02-13

**Authors:** Mahmoud Abdulbasser Sayed, Rania M. El-Sherif, Amira Ismail, Ahmed Essam Abou Warda, Amany R. Mohamed, Ahmed A. El-Sherif

**Affiliations:** 1 Department of Critical Care, Teacher’s Hospital, Cairo, Egypt; 2 Department of Critical Care Medicine, Faculty of Medicine, Cairo University, Cairo, Egypt; 3 Department of Clinical Pharmacy, Faculty of Pharmacy, October 6 University, Giza, 12585 Egypt; 4 Department of Physiotherapy, Cairo University, Cairo, Egypt

**Keywords:** Low-level laser therapy, Acupuncture, Left ventricular systolic heart failure, TDI, Nitric oxide, 6MWT, NYHA class

## Abstract

**Background:**

Low-level laser therapy (LLLT) is a promising noninvasive physiotherapeutic approach that has been demonstrated to improve cardiac performance. This study aimed to assess the impact of low-level laser therapy on cardiac functions and clinical status in patients with chronic left ventricular systolic heart failure who were not candidates for cardiac revascularization or resynchronization. A case series of 27 patients received a course of low-level laser physiotherapy, the clinical outcomes, echocardiographic parameters, and serum nitric oxide levels were evaluated before and after LLLT.

**Results:**

Of the total patients enrolled in the study, 21 (or 77.8%) were male, with a mean age of 57.7 ± 6.89 years. NYHA classification significantly improved after low-level laser therapy, 15 patients were in class III,12 were in class IV, and no one was in class II before laser therapy while after laser therapy; 25 patients shifted to class II, two patients were in class III with *P* < 0.001, Six-minute walk distance test was performed, and the results showed that the mean of 6MWT was less than 200 m (148.556 ± 39.092) before the study but increased to more than 300 after laser therapy (385.074 ± 61.740), left ventricular ejection fraction before laser therapy was 26 ± 7.5 while after laser therapy it became 30 ± 8.6 but diastolic function did not change after low-level laser therapy, the mean peak TR pressure was 40.0 ± 9.0 mmHg and 33.0 ± 7.0 before and after laser therapy respectively *P* < 0.001. A significant change was observed in NO level from 4.1 ± 1.4 IU/ml before laser therapy to 5.2 ± 1.7 IU/ml after laser therapy *P* < 0.001.

**Conclusions:**

Low-level laser therapy may add benefits to improve symptoms, clinical condition, and quality of life in patients with left ventricular systolic dysfunction, further studies are necessary to evaluate the changes in cardiac functions at a longer follow-up duration.

## Background

Low-Level Laser Therapy (LLLT) has emerged as a promising physiotherapeutic technique since it is noninvasive and relatively inexpensive. It has been shown that LLLT enhances cardiac function and myocardial contractility, decreases blood pressure, and improves myocardial, coronary, and aerobic reserves [1, 2]. This clinicofunctional efficacy was accompanied by modifications that were favorable to lipid metabolism, lipid peroxidation activity, antioxidant defence, hemocoagulation, and microcirculation [3].

Laser therapy has been proven to substantially reduce systolic, diastolic, and mean arterial pressure [4]. Moreover, submaximal cycling exercise was also found to have a favorable hypotensive effect, with no increase in diastolic arterial pressure and a decrease in total peripheral vascular resistance [5, 6]. The use of laser puncture allowed patients to use less hypotensive medications and allowed physicians to prescribe laser therapy regardless of the patient’s hemodynamic parameters [7].

Laser therapy has a cardioprotective effect, which makes it beneficial for patients with exertional angina and has positive effects on patients' overall health, as seen in a reduction in the incidence of anginal attacks and episodes of myocardial ischemia without pain, and this is accompanied by other beneficial changes in hemodynamic status [8, 9]. A substantial decrease in pain-free episodes of myocardial ischemia, which is a prognostically beneficial fact, is one indication that laser therapy had an impact on the relationship between painful and painless ischemia of the myocardium [10].

Nitric oxide (NO) may be photo-released from extra intracellular storage, such as nitrosylated hemoglobin and nitrosylated myoglobin, in addition to being photo-dissociated from Cox [11]. Furchgott first identified light-mediated vasodilation in 1968 while working on the nitric oxide project that would earn him the 1998 Nobel Prize [12, 13].

Furchgott's pioneering work was later expanded and validated by other investigators, who also demonstrated how light might affect the localized generation or release of NO and trigger vasodilation by way of the impact of NO on cyclic guanine monophosphate (cGMP) [14]. According to these studies, lighting devices with suitable designs could serve as efficient, noninvasive therapeutic agents for individuals who would benefit from elevated NO levels [12, 15].

Based on the aforementioned rationale, we hypothesized that cardiac functions might be improved after LLLT; therefore, we sought to compare the echocardiographic parameters, clinical characteristics, and serum nitric oxide levels in patients with chronic systolic heart failure before and after LLLT.

## Methods

### Study design and setting

A case series of 27 consecutive adult patients with chronic systolic heart failure secondary to ischemic heart disease was conducted at Critical Care Department, Cairo University Hospitals between May 2017, and December 2019. The Declaration of Helsinki's guidelines were followed during the study. The Ethics Committee and institutional review boards of both the Critical Care Medicine Department and Faculty of Pharmacy, October 6 University examined and approved the study protocol and informed consent form. All patients who participated in the trial provided written fully informed consent.

### Study population and follow-up

Patients who were older than 18 years old were diagnosed with systolic heart failure based on clinical presentation and confirmed on echocardiography. Patients with a left ventricular ejection fraction (LVEF) < 45% and New York Heart Association (NYHA) classes III and IV, Patients who were not candidates for CRT or revascularization as well as those in NYHA Classes IV or III or II who were receiving optimal medical care and had been hospitalized for HF during the previous 12 months were included in the study. Patients who had been scheduled for (CABG) or percutaneous coronary intervention (PCI) during the previous 90 days or CRT were excluded. Other exclusion criteria included pregnancy, malignancy, thyroid illness, epilepsy, LBBB with QRS 130 ms, and contraindications to laser therapy. All patients were subjected to optimization of the medical treatment for three months before the study. Each patient received 10 sessions of low-level laser therapy. The measurement parameters were directly before the first session and following the last session. Blood samples were withdrawn before and after the laser therapy for measuring serum nitric oxide by the enzyme-linked immunosorbent assay (ELISA). NYHA classification was assessed for all patients before and after the study. All patients underwent the six-minute walk test (6MWT) both before and after the research, with standardized instructions and encouragement. Patients were told to move as quickly as they could while walking as far as they could. They were informed that, if required, they might slow down or even halt. As with the hallway test, the patient was allowed to rest or stop at any time before or during the treadmill walk test. The walk test was also stopped if the patient experienced chest pain, unbearable dyspnea, cramps, disorientation, diaphoresis, or pallor.

### Echocardiography measurements

Using the transthoracic cardiac probe (X5-1) with tissue Doppler capability from the Philips iE33 machine, echocardiographic evaluation was performed both before and after the investigation. To evaluate the systolic and diastolic function of the left side of the heart, 2-D echocardiography, and tissue doppler imaging (TDI) are used to assess the left ventricular function. Systolic function was evaluated using geometry (modified Simpson method), the operator tracing the end-diastolic and end-systolic volumes, and the machine then automatically calculated the ejection fraction on the software using the formula: ejection fraction = (EDV − ESV)/EDV. Septal E/e' was used to assess diastolic function in addition to assessing E wave mitral deceleration time. Mitral regurge was evaluated by the regurgitant jet area/left atrial region, vena contracta, and mitral regurgitant jet area. Vena contracta greater than 5.0 mm regarded severe, 3–5 mm moderate, and less than 3.0 mm considered mild mitral regurgitation [16], whereas more than 40% considered severe mitral regurgitation, 20–40% considered moderate, and less than 20% considered mild mitral regurgitation. Applying a continuous wave doppler cursor in alignment with tricuspid regurgitation allowed for the measurement of the peak tricuspid regurgitation pressure, which was 4V^2^. All echocardiography parameters were performed by one cardiologist.

### Laser acupuncture therapy procedure

**Fig. 1:**
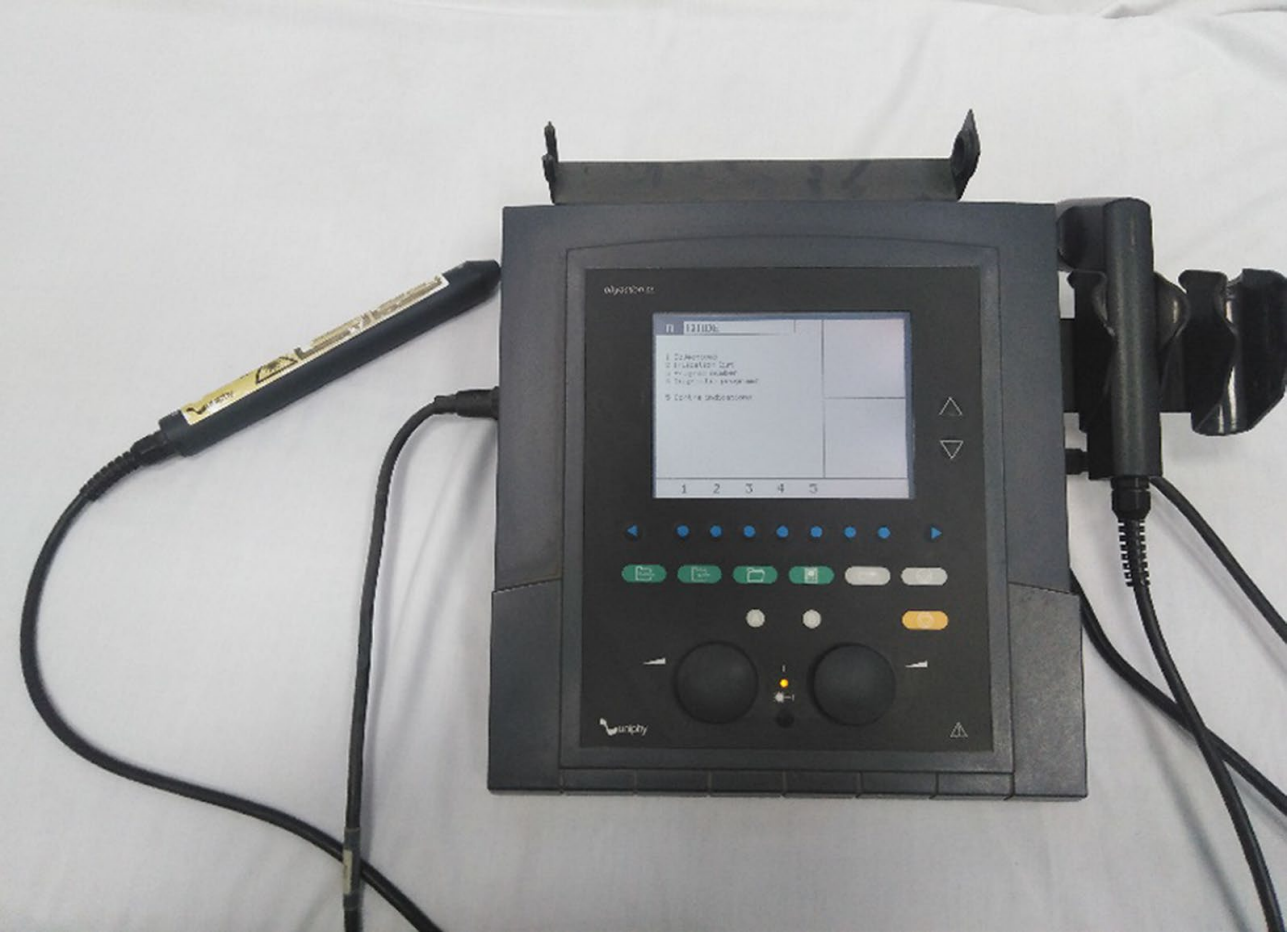
Laser acupuncture machine phyaction (CL)

**Fig. 2:**
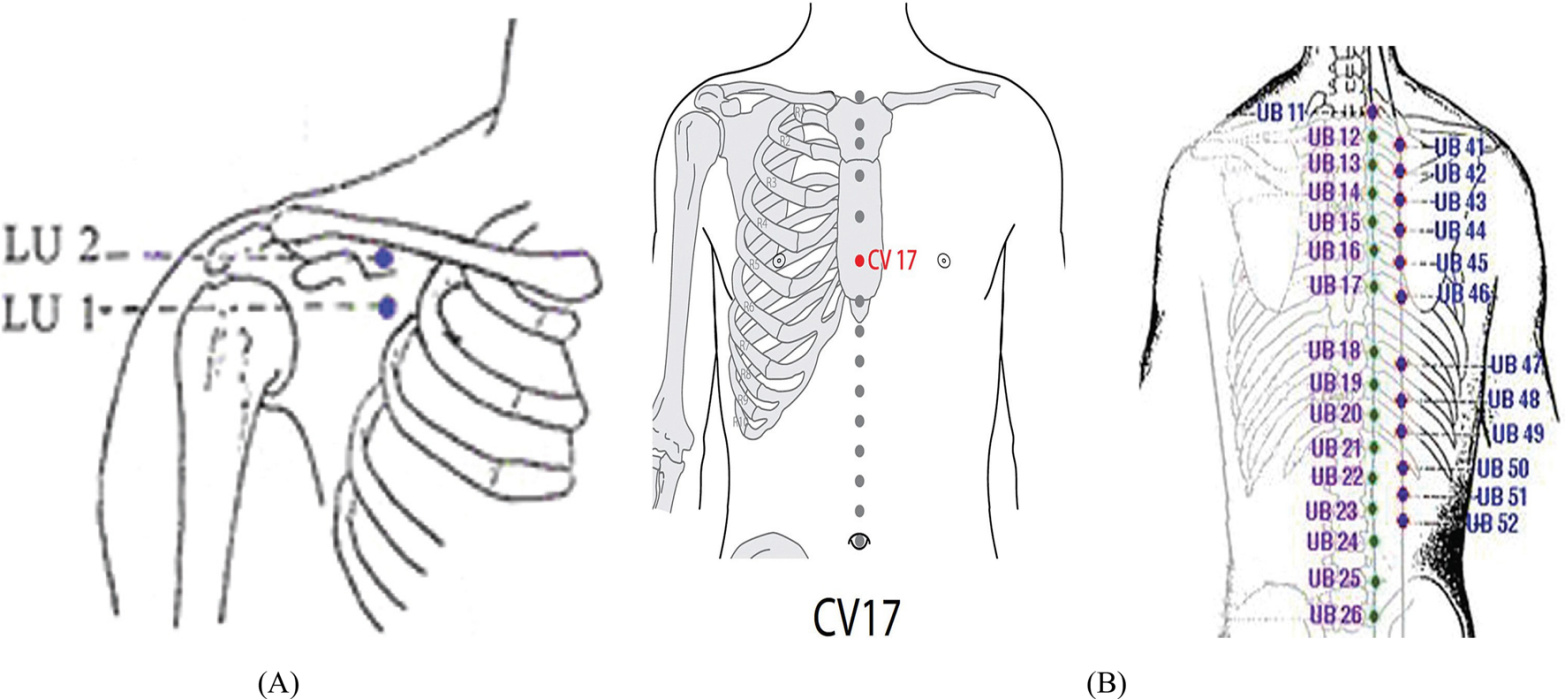
**A**, **B** Low level laser acupuncture points as illustrated; laser beam emits from the probe which is directed to the acupuncture points for one to two minutes at each point

Patients were subjected to low level laser therapy using a therapeutic unit (Phyaction CL) with wavelength 905 nm, output 5–20 MW, laser beam spot size 0.785 cm2, energy density 91 J/cm^2^, and energy delivered 28 J, and frequency 500HZ (Fig. 1). The laser wavelength was chosen accordingly significant effects of LLLT were previously reported [17]. Laser probe was placed on the intercostal space corresponding to the myocardium lesion both anteriorly and posteriorly on the chest wall and arm with standardized laser acupuncture points of application. The patient laid in the supine position, with hip and knee joints flexed and feet rested on the bed in a relaxed position. The site of application of laser therapy must be on clean skin. Each acupuncture point was stimulated with a laser for 60 s, once daily, with the frequency of five weekly sessions for two successive weeks. Acupuncture points are anterior chest wall LU1, LU2, CV17, (Ren17), posterior chest wall from a prone or setting position of the patient UB13, (BL13), UB 17(BL17), and arm UL5, UL7366 (Fig. 2A, B).

### Statistical analysis

Categorical data were presented as numbers and proportions. Paired categorical data was compared using the Mcnemar test. Normality testing was conducted using the Shapiro-walk test. Continuous variables that were normally distributed were presented in terms of mean and standard deviation and compared using paired t-test. The median and interquartile range were shown for non-normally distributed variables. All statistical tests with a value of *P* < 0.05 were considered statistically significant. All statistical analyses were done with Statistical Package for Social Sciences (SPSS), version 26 (SPSS Inc, Chicago, IL).

## Results

### Demographics and baseline characteristics

A total of 67 patients were assessed for eligibility during the study period. Where 37 of them were not meeting the inclusion criteria, and 3 of them were dropped from the trial because of technical issues with the laser acupuncture machine during regular sessions. In the overall sample, 21(77.8%) of the patients were males while 6 (22.2%) were female, the mean age was 57.7 ± 6.89 years. Of the 25 patients had a non-viable myocardium as per dobutamine echocardiography, and 8 patients refused revascularization. 19 (70.3%) of 27 patients were smokers, and 15 of them were diabetics for more than 10 years. 6 (22.2%) patients were hypertensive for years. Also, all patients were in sinus rhythm with a narrow QRS complex. All baseline characteristics of patients are shown in Table 1.

**Table 1:** Demographics and baseline characteristics of the participants

Baseline characteristics	*n* = 27
Age (years), mean ± SD	57.7 ± 6.89
Gender, male *n* (%)	21 (77.8%)
Body mass index, mean ± SD	23.6 ± 3.4
Systolic blood pressure, mmHg, mean ± SD	119.37 ± 9.39
Heart rate, bpm, mean ± SD	71.81 ± 5.04
NYHA classification	
NYHA III, *n* (%)	15 (55.6%)
NYHA IV, *n* (%)	12 (44.4%)
6 MWT (m), mean ± SD	148.56 ± 38.54
Medical history	
Hypertension, *n* (%)	6 (22.2%)
Diabetes mellitus, *n* (%)	15 (55.5%)
Atrial fibrillation, *n* (%)	4 (14.8%)
Family history with HF, *n* (%)	2 (7.4%)
Smoker, *n* (%)	19 (70.3%)
Baseline medication	
ACE inhibitor, *n* (%)	17 (62.9%)
ARB, *n* (%)	9 (33.3%)
β-Blocker, *n* (%)	22 (81.4%)
Sacubitril/valsartan, *n* (%)	1 (3.7%)
Aldosterone antagonist, *n* (%)	11 (40.7%)
Diuretic, *n* (%)	6 (22.2%)
Statin, *n* (%)	22 (81.4%)
Echocardiographic parameters	
Diastolic dysfunction grade I, *n* (%)	2 (7.4%)
Diastolic dysfunction grade II, *n* (%)	3 (11.1%)
Diastolic dysfunction grade III, *n* (%)	22 (81.5%)
LVEF%, mean ± SD	25.89 ± 7.4
End-systolic volume mL, mean ± SD	126.8 ± 51.3
End-diastolic volume mL, mean ± SD	190.7 ± 64.5
*E*, cm/s, median (IQR)	100 (80–120)
*A*, cm/s, median (IQR)	25 (30–50)
*E*/*A*, median (IQR)	3.0 (2.3–3.66)
*E*/*E*′ (cm/s), mean ± SD	17.48 ± 5.21
Regurge volume/LA volume, mean ± SD	27.05 ± 9.31
Vena contracta (mm), mean ± SD	4.72 ± 1.27
PASP mmHg, mean ± SD	39.56 ± 8.62
NO level IU/ml, mean ± SD	4.05 ± 1.41

*NYHA*—New York Heart Association, *6 MWT*—six-minute walk test, *LVEF*—left ventricle ejection fraction, *PASP*—pulmonary artery systolic pressure, *NO*—nitric oxide, *SD*—standard deviation

*Statistically significant

### Clinical parameters assessment

NYHA classification and six-minute walk distance were monitored before and after LLLT, regarding NYHA classification, 15 (55.5%) patients were in class III, 12 (44.4%) were in class IV, none of the patients in class II before laser therapy while after laser therapy; 25 (92.5%) patients shifted to class II, 8 (29.6%) patients were in class III with *P* < 0.001 denoting a statistically significant clinical improvement as shown in Table 2.

**Table 2:** Clinical assessments of the study participants

Time	Pre-LLLT	Post-LLLT	*p* value
Number of patients in NYHA class II	0 (0.00%)	25 (92.59%)	< 0.001*
Number of patients in NYHA class III	15 (55.56%)	8 (7.41%)	
Number of patients in NYHA class IV	12 (44.44%)	0 (0.00%)	
6MWT (meter), mean ± SD	148.556 ± 39.092	385.074 ± 61.740	< 0.001*
Min–max	110–234	234–470	

*LLLT*—low-level laser therapy, *NYHA*—New York Heart Association, *6 MWT*—six-minute walk test, *SD*—standard deviation

*Statistically significant

Regarding the six-minute walk distance, the mean was less than 200 m before the study (149 ± 39.0 m) that was changed to more than 300 after laser therapy (385.074 ± 61.740) with statistically significant *P* < 0.001 denoting also significant clinical improvement as in Table 2.

### Echocardiographic parameters

Left ventricular ejection fraction was detected by the Simpson method; the mean percentage for ejection fraction was 25.89 ± 7.4 before laser therapy which changed to 30 ± 8.6 after laser therapy with a statistically significant difference *P* < 0.001 (Table 3).

On the other hand, there was no change in diastolic function before and after laser therapy, 22 (81%) of the patients were in diastolic dysfunction grade three before LLLT, only 2 (7.4%) patients showed diastolic dysfunction grade one and 3 (11.1%) of them were in diastolic dysfunction grade two after LLLT. The change in the ratio of septal E/e was assessed and the mean was 17.0 ± 5.0 before laser therapy which became 18.0 ± 5.0 after laser therapy denoting no significant change with both measures *P* = 0.913  as in Table 3.

On the basis that regurgitant area/left atrial area less than 20% considered mild, 20–40% moderate, and more than 40% severe; our study revealed that 20 (74%) patients were in moderate mitral regurgitation (MR), 3 (11%) patients were in severe mitral regurgitation and 4 (15%) patients were in mild regurgitation before the study, while after laser therapy, 19 (7.03%) patients were in moderate MR, 2 (7.4%) patients were in severe MR and 6 (22.2%) patients were in mild MR.

The mean regurge area/left atrial area was 27.0 ± 9.0 before laser therapy and 26.0 ± 9.0 after laser therapy with non-significant *P* = 0.357 denoting moderate mitral regurgitation that wasn’t affected by laser therapy. Another parameter was assessed to detect the severity of mitral regurgitation; the regurge vena contracta (V.C), Our results showed a mean V.C of 4.7 ± 1.3 and 4.7 ± 1.8 before and after laser therapy subsequently denoting moderate mitral regurgitation (3–5 mm) with no significant *P* = 0.284 as in Table 3.

The mean of Peak tricuspid regurge pressure was 40.0 ± 9.0 mmHg before laser therapy and 33.0 ± 7.0 mmHg after laser therapy with a statistically significant difference between both measures *P* < 0.001 as shown in Table 3.

**Table 3:** Echocardiographic parameters assessment of the study participants

Parameter	Pre-LLLT	Post-LLLT	*P* value
LVEF%, mean ± SD	25.889 ± 7.511	29.963 ± 8.596	< 0.001*
Min–max	11–39	14–53	
*E*/*e* (cm/s), mean ± SD	17.487 ± 5.288	17.552 ± 5.510	0.913
Min–max	7–27	7–29	
RA/LA area %, mean ± SD	27.056 ± 9.451	26.259 ± 8.751	0.357
Min–max	13–50	10–48	
Vena contracta (mm), mean ± SD	4.7 ± 1.3	4.7 ± 1.8	0.284
Min–max	3–7	3–7	
Peak TR pressure (mmHg), mean ± SD	40.0 ± 9.0	33 ± 7.0	< 0.001*
Min–max	27–61	20–49	

*LLLT*—low-level laser therapy, *SD*—standard deviation, *LVEF*—left ventricle ejection fraction, *RA/LA*—regurgitation area/left atrial area, *TR*—tricuspid regurgitation

*Statistically significant

### Serum nitric oxide level

Nitric oxide levels measured before and after laser therapy, nitric oxide level revealed statistically significant with a mean of 4.1 ± 1.4 (IU/ml) before laser therapy and 5.2 ± 1.7 (IU/ml) after laser therapy *P* value < 0.001.

## Discussion

To the best of our knowledge, the current study is the first clinical study to evaluate the clinical outcomes and echocardiographic parameters in patients with left ventricular systolic failure after low-level laser therapy, a topic that has not been thoroughly addressed in the literature.

Even though the therapeutic mechanism of LLLT on cardiac muscle is still not fully known, numerous studies have suggested that it may have potent anti-inflammatory effects in the clinical setting [18]. LLLT can suppress circulating cytokines levels, in particular, tumor necrosis factor-α (TNF-α) and interleukin (IL)-6, these cytokines can promote progressive left ventricular (LV) dysfunction, LV remodeling, and cardiomyopathy [19]. Another explanation for how LLLT improves cardiac function is that it does by increasing the expression of vasoactive peptides and enhancing the production of nitric oxide (NO), which would reduce the acute inflammatory response in the myocardium and has a beneficial impact on LV function but at low concentrations [20, 21].

The findings of the current study showed an improvement in LV function after LLLT, which may be explained by the release of nitric oxide, which causes peripheral vasodilatation, reduces peripheral resistance, and then improves cardiac output [22]. Also, may be explained by an improvement in cardiac muscle performance due to the increased intake of energy provided by the increased aerobic metabolism stimulated by LLLT [9, 23]. Our findings were supported by several experimental studies that demonstrated the impact of low-level laser therapy on the infarction size, geometry, and LV function following myocardial infarction. These studies exhibited a decrease in infarct size and an improvement in cardiac performance [21]. Indeed, Low-level laser therapy was applied in a single, small-sample-size clinical study that reduces cardiac cellular damage and accelerates cardiac performance in patients recovering from coronary artery bypass grafting [24].

The New York Heart Association (NYHA) classification and the 6-min walking test (6MWT) have been widely supported by medical societies around the world and have been used in clinical studies showing the beneficial effects of various medications on mortality and morbidity in patients with chronic systolic failure [25]. In our study, we use the NYHA classification and the six-minute walk test to assess the clinical impact of LLL therapy in patients with chronic systolic failure. The results showed that low-level laser therapy was effective in reducing NYHA and 6-mwt. In contrast, Bublitz et al. conducted a pilot trial using LLLT on hospitalized patients with decompensated Heart Failure who then underwent a 6-min walking test, the total distance during this functional test was unaffected by 28 J of LLLT or 100 MW power [26]. However, clinical trials and a meta-analysis established that functional performance would be varied based on the population investigated and the laser dose applied [27, 28].

Unfortunately, our study found no statistically significant difference in left ventricular diastolic function. The lack of a beneficial effect of LLLT on diastolic function. Patients in this group are not a homogenous group. It's caused by a wide variety of disorders that may require individualized treatment [29] Additionally, the SGLT-2 inhibitor is currently the only type of drug or treatment that has significantly improved this group of patients [30].

It is important to note that mitral regurgitation significantly increased end-diastolic and end-systolic volumes of the left ventricle, indicating adverse cardiac remodeling and worse systolic function [31]. In this context, our study evaluated mitral regurgitation through the assessment of vena contracta width and regurgitation area to the left atrial area before and after laser therapy, but without any significant change. This may be due to the short follow-up period.

Peak tricuspid regurgitation pressure is a critical measurement for evaluating pulmonary artery systolic pressure and an important parameter in detecting the severity and follow-up of patients with left ventricular systolic heart failure [32]. Our study revealed a statistically significant difference between the mean peak TR pressure measurements before and after laser therapy; this finding was supported by a study conducted by Sayed et al. that examined the impact of LLLT in patients with chronic obstructive pulmonary disease and evaluated the echocardiographic of the right ventricular functions. It was discovered that low-level laser therapy did not lead the PASP to increase [33].The importance of NO for the cardiac function is still hotly debated, and a number of mechanisms of inotropic effects of low-level laser therapy. NO have been clarified in experimental studies. These include the cGMP-mediated inhibition of phosphodiesterase and subsequently increased cAMP [34, 35], direct activation of adenylyl cyclases [36], and enhanced excitation–contraction coupling by S-nitrosylation [37] or by increasing contractile calcium responsiveness [38]. Nitric oxide levels in the patients who were enrolled on our study were assessed, and it was found that they were statistically significant both before and after laser therapy. These findings were corroborated by experimental studies which showed an increase in NO levels in cardiac muscle that was associated with a cardioprotective effect [39].

Some limitations should be taken into account. To start, a small sample of participants were enrolled in our study since it was difficult for patients with persistent systolic heart failure to attend appointments and because it was challenging for them to attend physiotherapy sessions regularly. Second, we were unable to evaluate regurgitant volume using PISA and EROA measurements or assess LV systolic function with GLS analysis since our echocardiogram did not support this software. Lastly, the short duration of follow-up.

## Conclusions

Low-level laser therapy is regarded as a beneficial modality which may improve symptoms, clinical condition, and quality of life in patients with left ventricular systolic dysfunction, it was challenging to come to any firm conclusions about the improvement of left ventricular ejection fraction since there was no control group and a limited sample size. This may be just a coincidence, or a correlation, further studies and long-term follow-ups are necessary to evaluate the changes in cardiac functions induced by LLLT.

## Data Availability

Applicable upon request.

## Abbreviations

LLLT: Low-level laser therapy
CRT: Cardiac resynchronization therapy
NO: Nitric oxide
cGMP: Cyclic guanine monophosphate
cAMP: Cyclic guanine monophosphate
LVEF: Left ventricular ejection fraction
TDI: Tissue Doppler imaging
NYHA: New York Heart Association
CABG: Coronary angiography bypass graft
PCI: Percutaneous coronary intervention
6 MWT: Six-minute walk test
ELISA: Enzyme-linked immunosorbent assay
EDV: End diastolic volume
ESV: End systolic volume
LBBB: Left bundle branch block
PASP: Pulmonary artery systolic pressure
RA/LA: Regurgitation area/left atrial area
MR: Mitral regurgitation
TR: Tricuspid regurgitation
TNF-α: Tumor necrosis factor-α
IL-6: Interleukin-6
PISA: Proximal isovelocity surface area
EROA: Effective regurgitant orifice area
GLS: Global longitudinal strain
